# Hospitals and Dangerous Drugs

**Published:** 1924-10

**Authors:** E. W. E. Holderness

**Affiliations:** Home Office, Whitehall


### Hospitals and Dangerous Drugs.

Sir,—I am directed by the Secretary of State to say that his attention has been called to the fact that the wording of the conditions attached to the Order made on August 15, 1921, under the Dangerous Drugs Act, 1920, for the exemption of certain hospitals, etc., from the Dangerous Drugs Regulations, in certain respects imposed restrictions on medicines which did not themselves come within the scope of the Regulations. In order to make clear that such substances are not to be regarded as subject to the conditions attached to the Order, the Secretary of State has added to both Schedule I. and Schedule II. a new condition as follows:—"Nothing in this Schedule shall affect any medicine or other substance to which the Regulations do not apply."

The alteration has been effected by the issue of a new Order (copy enclosed) entitled the "Dangerous Drugs (Hospital General Exemption) Order (1924)," which replaces the Order of August 15, 1921. Apart from the new conditions referred to above, this Order differs only in verbal points from the former Order, but it should nevertheless be in the possession of all hospitals affected by its terms.

E. W. E. Holderness

Home Office, Whitehall.

